# *Ms44*-SPT: unique genetic technology simplifies and improves hybrid maize seed production in sub-Saharan Africa

**DOI:** 10.1038/s41598-024-83931-1

**Published:** 2024-12-30

**Authors:** Sarah Collinson, Jill E. Cairns, Lucia Ndlala, Benjamin Deonovic, Dickson Ligeyo, Marc Albertsen, Walter Chivasa, Kingstone Mashingaidze, Michael S. Olsen, Berhanu T. Ertiro, Boddupalli M. Prasanna

**Affiliations:** 1 Corteva Agriscience, 7000 NW 62Nd Ave, Johnston, IA 50131 USA; 2 International Maize and Wheat Improvement Center (CIMMYT), 12.5 KM Peg, Mazowe Road, Mount Pleasant, MP163, Harare, Zimbabwe; 3 Agricultural Research Council (ARC)- Grain Crops Institute, Private Bag X1251, Potchefstroom, South Africa; 4 Kenya Agricultural and Livestock Research Organization (KALRO), P.O. Box 450 30200, Kitale, Kenya; 5 International Maize and Wheat Improvement Center (CIMMYT), United Nations Avenue, Gigiri, PO Box 25171, Nairobi, Kenya

**Keywords:** Detasseling, Nuclear genetic male sterility, Hybrid maize, Hybrid seed production, Plant genetics, Plant reproduction

## Abstract

Hybrid maize seed production in Africa is dependent upon manual detasseling of the female parental lines, often resulting in plant damage that can lead to reduced seed yields on those detasseled lines. Additionally, incomplete detasseling can result in hybrid purity issues that can lead to production fields being rejected. A unique nuclear genetic male sterility seed production technology, referred to as *Ms44*-SPT, was developed to avoid hybrid seed loss and to improve the purity and quality of hybrid maize production. Hybrid seed yield reduction following detasseling can be attributed to leaf loss. Our analyses showed an average 2.9 leaves are lost during the detasseling process, resulting in a seed yield reduction of 14.0%. These findings suggest that deploying the *Ms44*-SPT technology would avoid this seed yield loss. By simplifying hybrid production and increasing seed yields, *Ms44*-SPT could help drive hybrid replacement, providing smallholder farmers with better access to improved hybrids.

## Introduction

A steady stream of incrementally improved varieties has been an important strategy to increase cereal yields in an increasingly variable climate^1,2^. New maize hybrids offer yield gains to farmers, particularly in stress prone environments^3–5^. Over the past two decades there has been extensive investment in strengthening national and regional maize breeding programs in sub-Saharan Africa (SSA)^6^. The estimated area planted with new stress tolerant maize hybrids across eight countries in eastern and southern Africa (ESA) increased from 1.4 million hectares (M ha) in 2016 to 4.9 M ha in 2021 to 7 M ha in 2023^7,8^. The average area-weighted age of maize hybrids provides a measure of the rate of hybrid replacement^9^. While the average area-weighted age of maize hybrids in ESA has reduced to approximately 10 years^8^, private seed companies often remain hesitant to continuously update their hybrid portfolio. Key assumptions of market-led strategies to improve the livelihoods of smallholder farmers through the adoption of improved maize hybrids are that: a) seed companies, and farmers, see the value in adopting new hybrids; and b) seed companies are willing and able to continuously introduce new hybrids whilst phasing out older/obsolete hybrids^10^.

Demand for improved genetics alone does not appear to be a sufficient driver for the private sector to continuously replace maize hybrids^11^. Replacing older hybrids is expensive, complex, and time-consuming for seed companies^2,12^. Seed companies need to cover the cost of new hybrid release requirements and produce seed of the new hybrid for commercialisation, demonstration, and marketing requirements, whilst in parallel continuing seed production of the older hybrid to be phased out. While in other regions of the world, farmers demand new maize hybrids and very short product life cycles (< 5 years)^13^, this is not the case in SSA. The combination of complexity, high cost and associated financial risk is a deterrent to continued maize hybrid replacement by the private sector, and likely accounts, in part, for the continued sale of old/obsolete hybrids^2^.

Since farmer demand and intense competition for market share are currently not sufficiently driving hybrid replacement strategies in SSA, alternative drivers are required to facilitate hybrid turnover. The emergence of new diseases or pests has been one such driver of crop varietal replacement in the region^14,15^. Additionally, technological innovations which provide direct economic benefits to seed companies could also help drive hybrid replacement^16,17^.

Hybrid maize seed is produced by cross-pollinating two genetically distinct parents in isolated seed production fields. The tassel is removed from female parent plants to prevent self-pollination (a process referred to as detasseling), thus ensuring only pollen from male parent plants fertilizes female plants. Three-way hybrids dominate the commercial maize market in SSA^8,18^; thus, two hybridization steps, and consequently two detasseling steps, are required. Manual detasseling often reduces hybrid seed production yields due to inadvertent removal of leaves below the tassel. The loss of one leaf (plus tassel) was previously estimated to reduce hybrid seed production yields by 2.6%, whilst the removal of four leaves reduced seed production yields by 25.4%^19^. Genetic male sterility systems offer an alternative approach in hybrid maize seed production, avoiding the need to detassel, reducing the risk of self-pollination^20^, and reducing yield loss during hybrid seed production. Cytoplasmic male sterility (CMS) has been used since the 1950s to produce hybrid maize seed^21^, although it does not work across all genetic backgrounds and is influenced by the environment^21^. CMS can also be difficult to use in producing three-way hybrids because two non-restoring male parents must be used, the first to increase male-sterile seed of the initial female parent, and the second to produce male-sterile seed of the single-cross female parent. A further complication is the need to use a fully fertility-restoring male parent in the production of the final three-way hybrid seed.

**Fig. 1:**
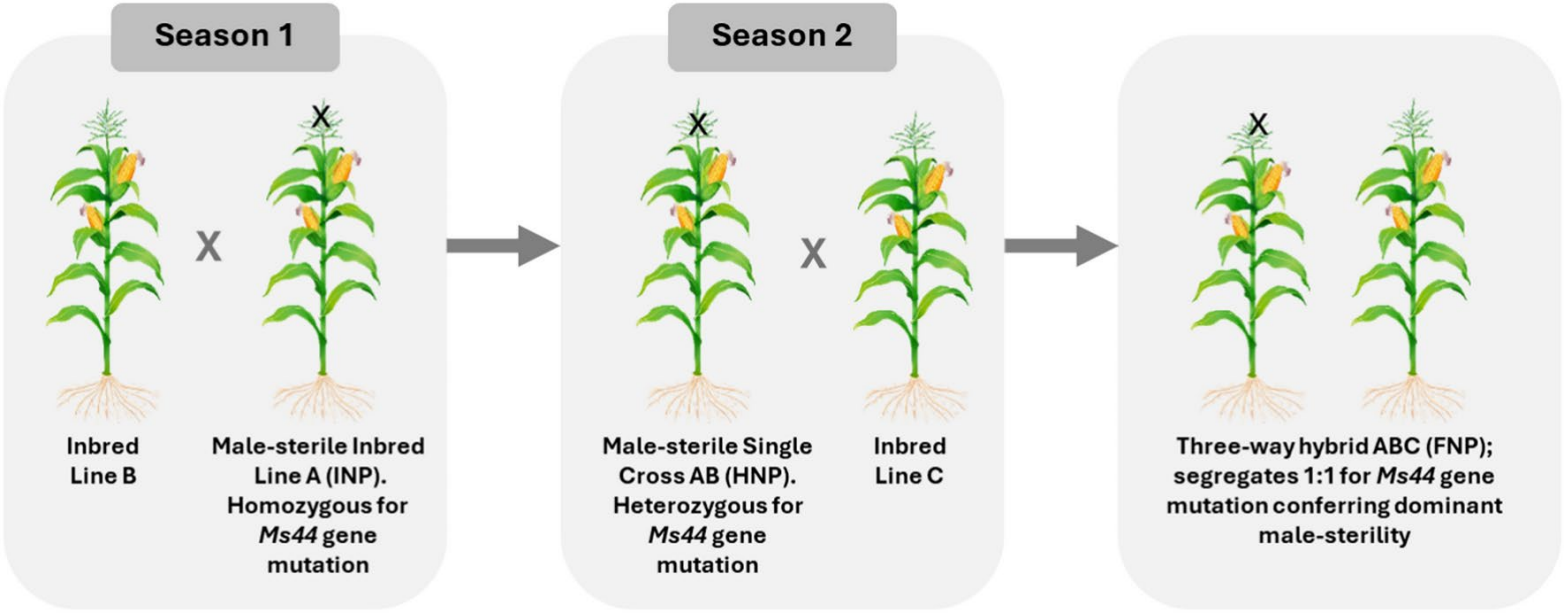
Three-way hybrid maize seed production using *Ms44*-Seed Production Technology (Ms44-SPTA). In Season 1, male and female parent inbred lines are planted in the seed production field to produce the single-cross. The non-transgenic female parent inbred line (Line A) has been converted to become homozygous for *Ms44*. It is referred to as inbred non-pollen producing (INP). The resulting single-cross, which is now heterozygous for *Ms44*, is referred to as heterozygous non-pollen producing (HNP). In Season 2, the HNP (single-cross female) and male inbred line (Line C) are planted in the seed production field to produce the final three-way hybrid. The resulting three-way hybrid segregates 50:50 for *Ms44* and is referred to as 50% non-pollen producing (FNP).

Nuclear genetic male sterility, which is more stable across genetic backgrounds and environments, has been used more recently to produce hybrid maize seed^22,23^. Corteva Agriscience® developed a nuclear genetic male sterility system, Seed Production Technology (SPT), based on the recessive maize male sterility gene, *ms45*, for hybrid maize production in the USA^24^. The seed production technology for Africa (SPTA) project later introduced the dominant maize male sterility gene, *Ms44*^18^. Because *Ms44* is dominant it is more suitable for three-way hybrid production as both the initial inbred female parent and the single-cross female parent are non-pollen producing (NPP). The need for detasseling during both hybridization steps, therefore, is eliminated (Fig. 1). Although a transgenic maintainer line is used to propagate the initial NPP female lines, the resulting inbred is not only non-pollen producing (INP), but it is also non-transgenic. This is a consequence of three linked components in the transgenic maintainer line. One component enables fertility of the maintainer line. A second component blocks inheritance of the entire maintainer construct through the pollen by pollen-specific expression of a gene that eliminates starch formation in pollen grains carrying the maintainer transgenic components^24^. A third component results in seed containing the maintainer transgenes to become reddish pink. To ensure that all progenies are non-GM, the initial female parent seed is run through a colour sorter as a final quality control step^22^. The transgenic maintainer components have gone through regulatory approval in both the USA and South Africa, ensuring they are safe for the public and the environment. The final hybrid maize seed sold to farmers, as well as the parental lines used by seed companies, is non-GM. Only the maintainer line is transgenic.

We estimated the seed yield benefit of using the *Ms44* nuclear genetic male sterility-based seed production technology, referred hereafter as *Ms44*-SPT, in hybrid maize seed production. To quantify seed production loss associated with leaf removal during detasseling we conducted a series of experiments using PP (pollen producing) and NPP single-cross female parent. In PP single cross female parents up to five leaves were removed. NPP single cross female parents were produced using the *Ms44*-SPT. To estimate actual leaf loss in seed production fields during the detasseling process, the number of leaves removed in commercial seed production fields were quantified. This allowed the average reduction in female seed production yields to be quantified.

## Results

### Maize seed yield reduction associated with leaf removal

**Fig. 2:**
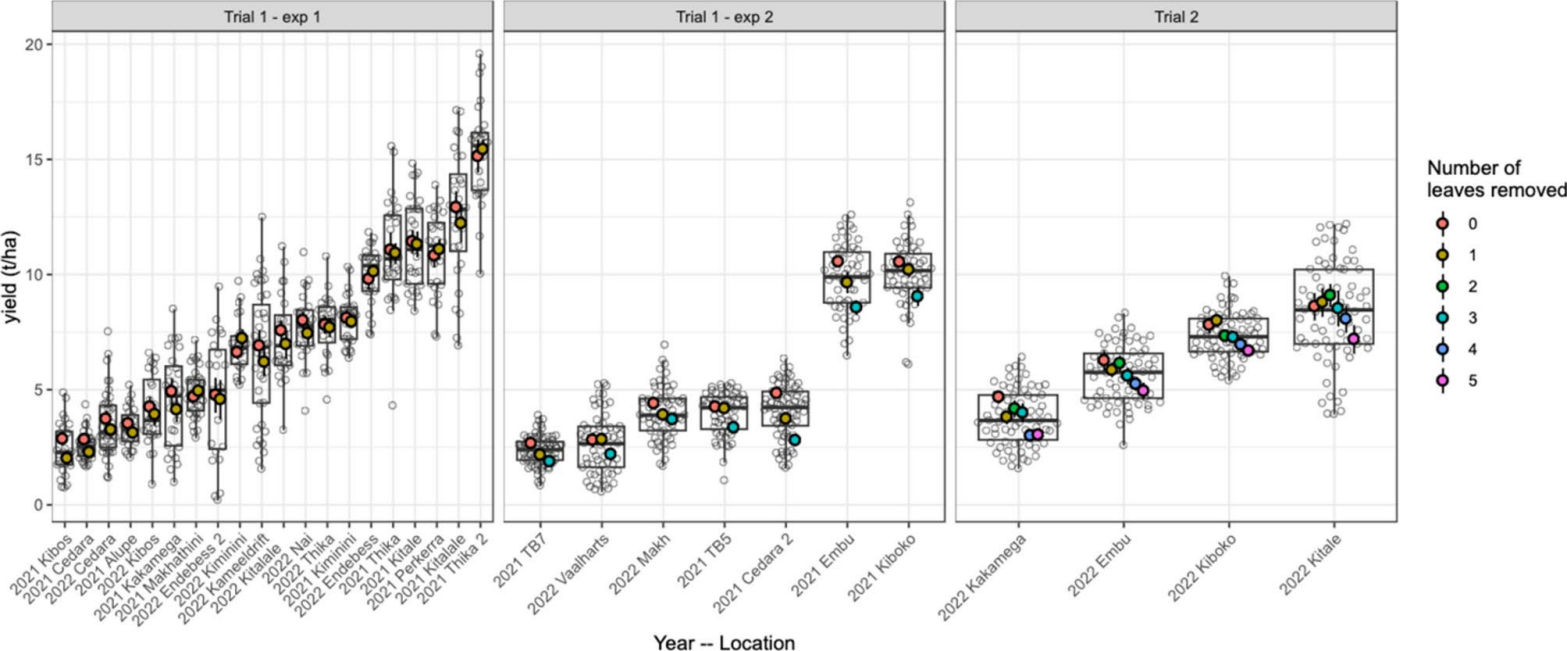
Average seed yield of Trial 1, Experiment 1 (Trial 1-exp 1) where one leaf was removed from the pollen-producing (PP) single-cross parents; Trial 1, Experiment 2 (Trial 1-exp 2) where one or three leaves were removed from the PP single-cross parents, and Trial 2 where one to five leaves were removed from the PP single-cross parents. Grey circles represent individual data points, and coloured circles represent the average seed yield at each location. The colour refers to the number of leaves removed.

**Fig. 3:**
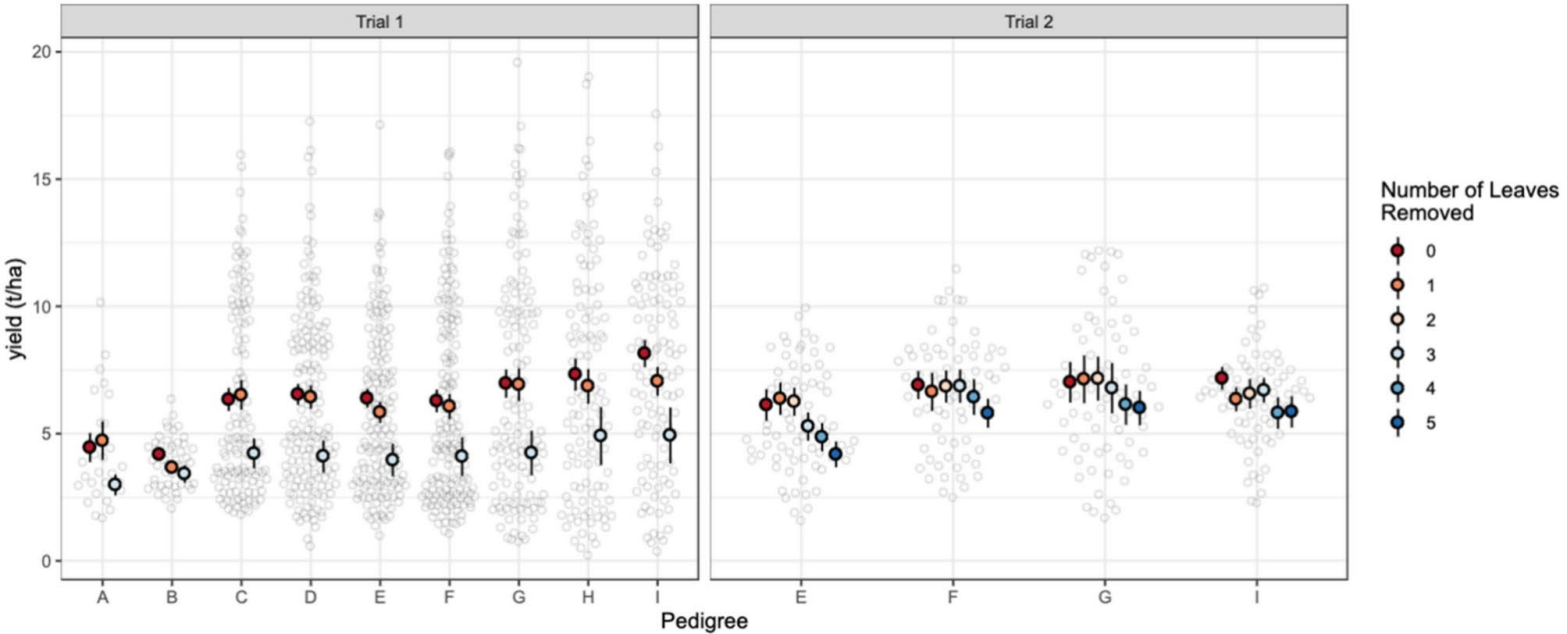
Average seed yield of Trial 1 (where one or three leaves were removed from the pollen-producing (PP) single-cross parents) and Trial 2 (where one to five leaves were removed from the PP single-cross parents) across pedigrees. Grey circles represent individual data points and coloured circles represent the average seed yield at each location. The colour refers to the number of leaves removed.

The first trial consisted of two experiments. In the first experiment (where one leaf was removed from PP single-cross parents), average seed yield across locations ranged from 2.4 to 15.3 t ha^−1^, at an average of 7.26 t ha^−1^ across locations (Fig. 2, Trial1-exp 1). In the second experiment where one or three leaves were removed, the average seed yield ranged from 2.35 to 10.1 t ha^−1^, at an average of 5.27 t ha^−1^ across locations (Fig. 2, Trial 1-exp 2). In the second trial (where one to five leaves were removed from the PP single cross parents), average seed yield across locations ranged from 3.78 to 8.37 t ha^−1^, at an average of 6.29 t ha^−1^ across locations (Fig. 2, Trial 2). Seed yield reduction was consistent across different entries (Fig. 3).

### Quantifying female seed loss associated with leaf removal during detasseling

**Fig. 4:**
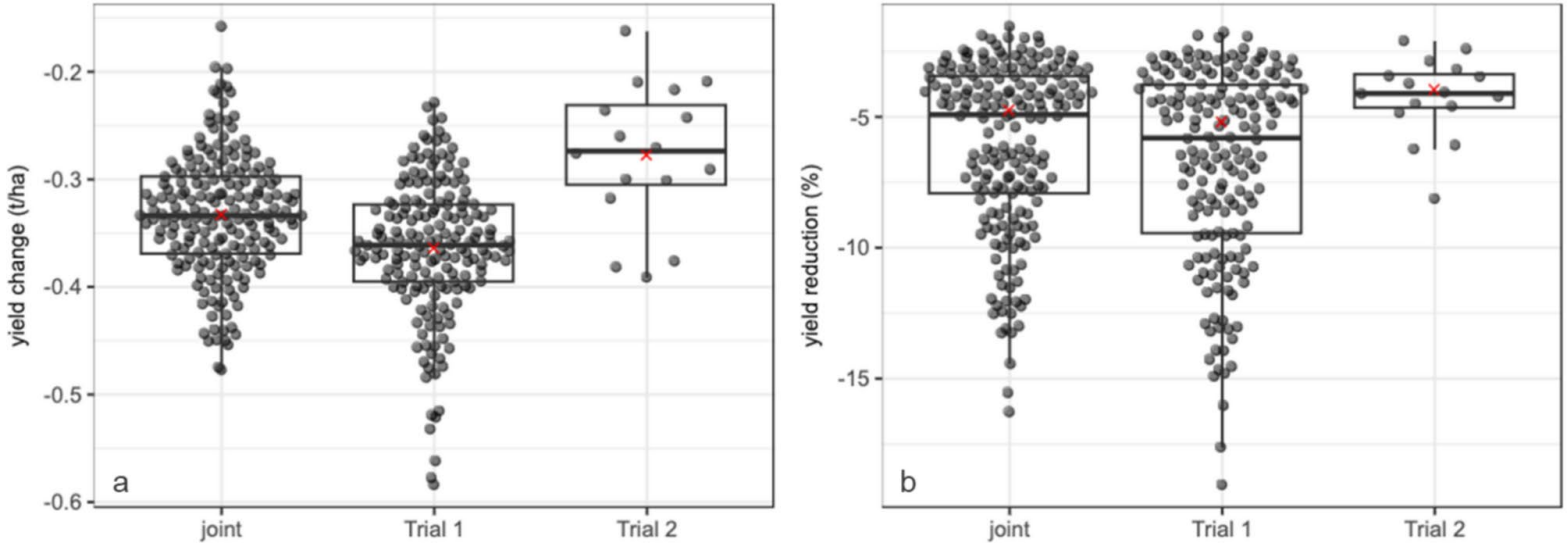
The (**a**) actual change in seed yield across all experiments (Joint) and (**b**) relative seed yield reduction per leaf removed in Trial 1, where one leaf (experiment one) and three leaves (experiment two) were removed from the pollen producing parent, and Trial 2 where up to five leaves were removed from the pollen producing parent and combined across all trials.

Overall, a significant reduction (n = 1009, p-value = 0.000999) in yield was observed per leaf removed in the first trial. The change in yield per leaf removed was estimated to be −0.37 (−0.58, −0.28) t ha^−1^ (Fig. 4), corresponding to a yield reduction of 5.2% per leaf. The change in yield per leaf removed in the second trial was also significant (n = 283, p-value = 0.000999). Yield was reduced by −0.28 (−0.36, −0.20) t ha^−1^ (Fig. 4) per leaf removed, with a corresponding yield reduction of 4.0%. Compared to the first trial, the yield reductions were attenuated in the second trial, an effect which is largely due to the smaller effects observed in this trial for removal of two and three leaves (Fig. 4).

**Fig. 5:**
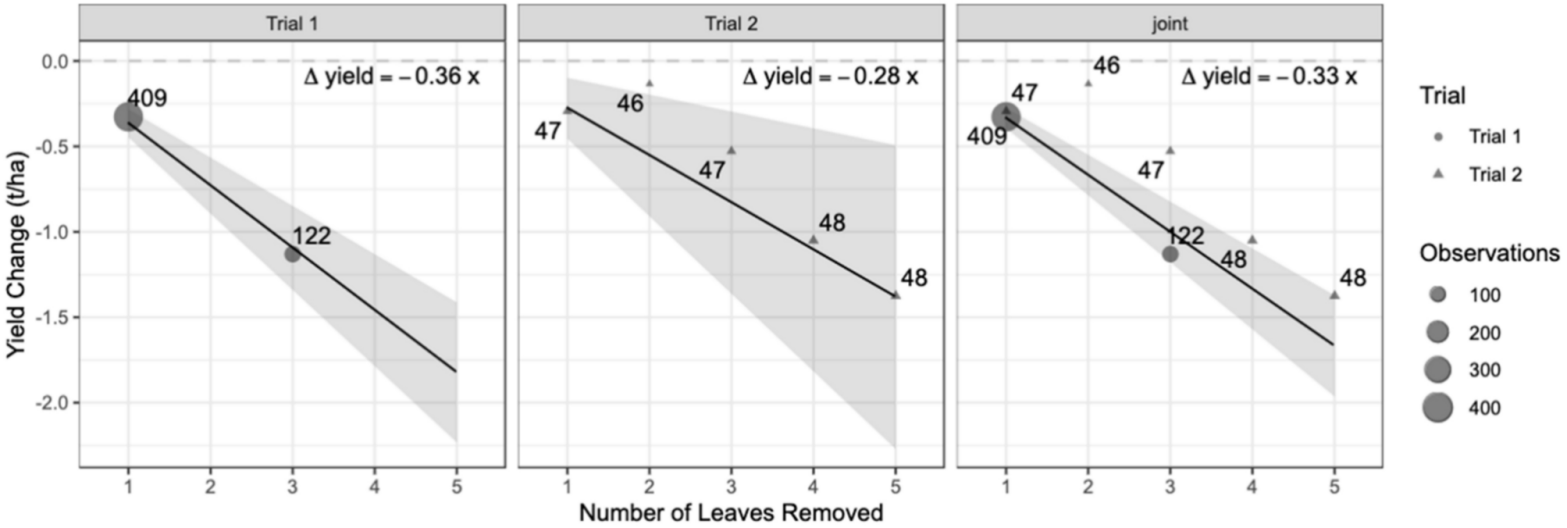
Estimated change on yield in Trial 1 (where one leaf (experiment one) and one or three leaves were removed from the pollen producing parent (experiment two)), and Trial 2 (where up to five leaves were removed from the pollen producing parent) and combined across all trials. The size of the bubble represents the number of observations. In the joint (combined) analysis, data from Trial 1 is indicated by a triangle and data from Trial 2 two is indicated by a triangle (and referred to as Joint).

When data was combined across the two trials in a joint analysis, we found a significant change (n = 1292, p-value = 0.000999) in yield of -0.33 t ha^−1^ (−0.49, −0.24), corresponding to a yield reduction of 4.8%, which is primarily driven by the effects observed in trial 1 due to the significantly larger sample size (Fig. 5). There was also a significant amount of variability observed in the effect of removing a single leaf. The estimated standard deviation of the random slope was 0.18, 0.10, and 0.13 in the models for trial one, trial 2, and the joint model respectively. When we incorporate the random effects for year-location and pedigree to obtain cluster specific effects, the estimates of the impact of removing one leaf range from −0.48 to −0.16 t ha^−1^ and the percent yield reductions ranged from −16.3 to −1.6% per leaf removed (Fig. 5).

### Seed size and weight

Less than 30.0% of PP and NPP seed was classified as round (Table 1). The largest seed grade in both PP and NPP was medium flat, accounting for 41.0 and 41.7% of the total seed, respectively. Within each class, there was no significant difference between the number of kernels produced in both the NPP and PP after adjusting for multiple comparisons using Holm-Sidak, except for large round grade (n = 680, p-value < 0.05). PP had more large round kernels. Similarly, for 100-kernel weight of each seed grade there were no significant difference after adjusting for multiple comparisons using Holm-Sidak, except for the large round grade (n = 680, p-value < 0.05). PP had a model estimated mean weight of 31.46 g and NPP 22.50 g. However, this was the smallest class, accounting for 1.8 and 2.4% of kernels in the NPP and PP respectively.

**Table 1:** Model average proportion and 100-kernel weight for each seed grade in non-pollen producing (NPP) and pollen-producing (PP) hybrids.

Trait	Pollen producing (n = 434)	Non-pollen producing (n = 246)	Difference	Lower 95% CI	Upper 95% CI	Adjusted p-value
Proportion						
Large flat	0.07	0.06	0.01	0.00	0.01	0.16
Large round	0.01	0.01	0.00	0.00	0.00	0.00
Medium flat	0.40	0.41	−0.01	−0.03	0.00	0.21
Medium round	0.07	0.07	0.00	−0.01	0.01	1.00
Small flat	0.23	0.24	−0.01	−0.02	0.01	1.00
Small round	0.05	0.05	0.00	−0.01	0.00	1.00
100-kernel weight (g)						
Large flat	40.93	42.43	−1.50	−5.66	2.62	1.00
Large round	31.46	22.50	8.96	3.41	14.03	0.00
Medium flat	41.64	41.78	−0.15	−3.27	2.68	1.00
Medium round	51.39	49.30	2.09	−3.57	6.61	1.00
Small flat	31.69	30.27	1.42	−1.69	4.06	1.00
Small round	32.04	31.39	0.64	−1.83	3.20	1.00

### Leaf removal in commercial hybrid maize seed production fields and estimated yield reduction associated with detasseling

**Fig. 6:**
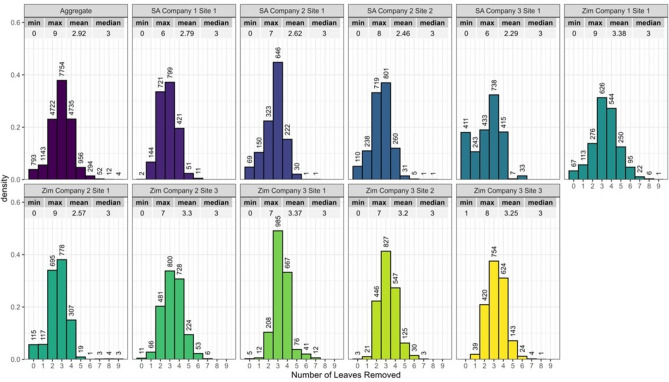
Frequency of the number of leaves removed along with the tassel during the detasseling process in commercial seed production fields in South Africa (SA) and Zimbabwe (Zim).

The distribution of top leaves removed during the detasseling process in three-way hybrid maize production was similar across nine commercial seed production fields (Fig. 6). Approximately 35.9% of removed tassels had three leaves attached. Over six percent of tassels (6.4%) had five or more leaves attached. A further 3.9% of tassels had no leaves attached. The highest number of leaves attached to the tassel was nine, although this only was observed on four tassels (Fig. 6). Across locations, an average of 2.81 leaves were removed during detasseling. Using the joint model described above the yield change in the typical location is predicted to be −0.97 t ha^−1^, which corresponds to a 14.0% reduction in yield. Considering the uncertainty in the fixed effect estimates, the random effects variability, and the distribution of leaves removed during detasseling in commercial hybrid maize seed production fields we simulated yields to obtain a predictive distribution of yield reduction. The mean yield reduction in these simulations was 17.0%, the median yield reduction was 12.6 and a 95% predictive interval of 62.0 to 0%.

## Discussion

This is the first documented study in Africa to estimate hybrid maize seed production loss associated with manual detasseling and the impact that new genetic technology can have on improving and simplifying hybrid maize production. The detasseling process is estimated to reduce hybrid maize seed production by 14.0%, representing a significant yield and associated economic loss to seed companies. This is the equivalent of an additional 0.70 or 0.98 t ha^−1^ in maize seed production fields at an average potential seed yield of 5 or 7 t ha^−1^, respectively. The economic benefit of this additional hybrid maize seed production will vary by seed company and the conditions under which production occurs within a country. Most maize hybrids on the market in SSA are three-way hybrids; thus, this loss will be compounded with the two hybridization steps required to produce three-way hybrids. The use of a nuclear genetic male sterility-based hybridization platform that bypasses the need for manual detasseling in both hybridization steps would offer a significant seed yield benefit to seed companies. An additional economic benefit not captured in this study is the possible loss associated with contaminating pollen shed within a seed production field if manual detasseling is not perfect.

Over the past two decades there has been an influx of new companies entering the hybrid maize seed business space in SSA, especially in ESA^25–27^. However, many of the new seed companies lack expertise and experience in maize hybrid seed production. This has been one of the major challenges both in terms of obtaining economic seed yields and achieving the required genetic purity standards of certified seed. Contaminating pollen shed within a seed production field warrants discarding a large area around any female parent shedding pollen or even condemning entire field. In one commercial seed production site that was visited as part of this study, most female parent plants had started to shed pollen, and the entire production field was condemned. The two hybridization steps in conventional three-way hybrid production increases the probability of contaminating pollen shed occurring.

Hybrids developed using *Ms44* segregate 50:50 for PP and NPP plants and are classified as 50% non-pollen producing (FNP). FNP hybrids have been shown to have a yield advantage to farmers under low input conditions, providing an additional benefit to smallholder farmers^18^. FNP hybrids have previously been shown to have a higher kernel number^18,28,29^, 100-kernel weight^18,30^, and kernel length^30^ relative to PP hybrids. Both detasseling and NPP reduce apical dominance and enhance biomass partitioning to the ear. However, most pollen grains are already formed if the tassel is removed just before anthesis^31,32^. One of the differences between detasseled plants and *Ms44* male-sterile plants is that the pollen does not fully form in *Ms44* male-sterile plants, likely reducing the demand for photosynthate to produce starch-filled pollen grains; this may be one of the reasons for improved yield. Detasseling does increase light interception, although the benefits of this are a function of the tassel size. Unlike temperate maize which has undergone intensive indirect selection for reducing tassel size, in tropical maize, in general, this trait has not changed. The potential positive effect on grain yield that may result from the removal of tassels only in the PP entries was not determined in this study.

Detasseling is undertaken before the tassel protrudes to avoid contaminating pollen shed. Only 3.9% of tassels collected in seed production fields had no attached leaves confirming this is relatively rare. There were no significant differences in the number of kernels produced by NPP and PP plants, except for the smallest class which accounted for less than 3.0% of kernels. Seed sizing was only conducted in the trial with one leaf removed. It is possible that when a greater number of leaves were removed the proportion of each seed class changes; however, similar results were observed during the use of SPT with *ms45.* No significant differences in seed sizing between hybrids produced with *ms45* SPT relative to hybrids produced with detasseling were identified (Marc Albertsen, *personal communication*).

The use of CMS, which also mitigates the need to detassel the female parent plant in hybrid maize seed production, would likely provide a similar seed yield benefit to the private sector^23^. The inherent disadvantage in identifying a second non-restoring male parent required in using CMS in three-way hybrid production not only limits its use in three-way hybrid production, but also can limit the choice of germplasm diversity to produce the most heterotic combinations possible. Furthermore, a CMS-derived hybrid blended with a conventionally derived hybrid does not consistently provide a yield benefit to farmers, unlike *Ms44* derived hybrids.

Mechanical detasseling largely bypasses the need for contract labour, but it can result in greater damage to the top leaves^33^. Despite varying levels of experience and training across the commercial seed production fields and the different three-way hybrids being produced in this study, the distribution of leaves removed with the tassel during the manual detasseling process was relatively similar across locations. Theoretically, seed yield loss could be lowered by reducing the number of leaves removed during the detasseling process; however, this increases the potential for self-pollination and risks higher economic losses to seed companies due to the failure of the production field to meet certification standards. Labour is increasingly a limiting factor in agriculture in SSA^34–36^. Competing demands for contract labour, primarily due to tobacco production and artisanal gold panning, were found in this study to be a major constraint faced across commercial seed production fields in South Africa and Kenya. Competing demands for labour with a tobacco farm were responsible for an entire maize seed production field being condemned in this study. In a second maize seed production field, labour was only available at the weekend due to competing labour demands within tobacco production.

Despite the plethora of studies highlighting the potential benefit of single gene technologies to increase crop yields, there are significantly less studies confirming their impact within a target environment^37^. The yield benefit provided by FNP hybrids was the first example of a single gene technology conferring a significant yield advantage in smallholder farmers’ fields in SSA^18^. The results of this study provide confirmation of a second benefit of this single gene, by mitigating the significant seed yield loss associated with the detasseling process in seed production, and at the same time simplifying seed production for seed producers.

## Conclusions

The use of the *Ms44*-SPT sterility-based hybridization platform in hybrid maize seed production would increase seed production yields by 14.0%, whilst also improving the genetic purity and quality of the final hybrid. In the production of three-way maize hybrid seed, this benefit would be realised in both hybridization steps, although the benefit during single-cross female production has not been quantified. The availability of new female lines converted with *Ms44*-SPT would offer significant economic benefit to seed companies and may provide an inflection point in the innovation pathway of climate-resilient maize hybrids in SSA.

## Material and methods

### Effect of leaf removal on hybrid maize seed production

A total of 18 single-cross female maize hybrids were used in this study. The source of maize seeds was the International Maize and Wheat Improvement Center (CIMMYT). Nine of the single-cross female hybrids were produced using female lines converted with *Ms44*-SPT and thus were non-pollen producing (NPP) and did not require detasseling for three-way hybrid seed production. The remaining nine single-cross females were produced using the same female lines that had not been converted with *Ms44*-SPT. They were, therefore, pollen-producing (PP) and required detasseling for three-way hybrid seed production. Due to limited seed availability, the number of pairs of single-cross females varied across trials. The number of entries for each experiment are listed in (Table 2).

**Table 2:** Summary of trials conducted in South Africa and Kenya in 2021 and 2022. Trials used in seed grading are also highlighted.

Trial-exp	Year	Country	Number of leaves removed	Locations	Entries	Reps	Seed grading
Trial 1–exp 1	2020–21	South Africa	1	2	12	3	
	2021	Kenya	1	9	14	2	Yes
	2021–22	South Africa	1	2	14	3	
	2022	Kenya	1	7	12	2	Yes
Trial 1–exp 2	2020–21	South Africa	1 & 3	3	12	3	
	2021	Kenya	1 & 3	2	14	2	Yes
	2021–22	South Africa	1 & 3	2	14	3	
Trial 2	2022	Kenya	1 to 5	4	8	3	Yes

Two different trials were conducted during 2021–2022 to quantify the effect of tassel and leaf removal during the detasseling process in hybrid maize seed production (Table 2). In the first trial, at tassel emergence but before pollen shed, the tassel and one or three leaves nearest to the tassel were removed from PP single-cross females. One leaf removal was conducted at a total of 20 locations, with one or three leaf removal at seven locations. These locations were planted with four-row plots. One leaf was removed from two rows, and three leaves were removed from the other two rows (rows were adjacent but assigned randomly). Because the tassel in NPP single-cross females did not produce pollen, it was not removed. Similarly, no leaves were removed from NPP single-cross females. These trials were conducted at nine locations in South Africa and 18 locations in Kenya.

The first year of trials were run concurrently with the sampling to determine the actual number of leaves removed in commercial maize seed production fields during the detasseling process. This highlighted the need to establish seed yield loss associated with the removal of more than three leaves. In 2022 a second trial with five treatments was conducted at four locations in Kenya with eight entries. Upon tassel emergence but before pollen shed, the tassel along with one, two, three, four or five top leaves (nearest the tassel) were removed from PP single-cross females. Each treatment was in a two-row plot, and plots were nested by hybrid. In NPP single-cross females, neither the tassel nor any top leaves were removed.

All trials were irrigated, and optimum agronomic management was followed. Plots were 3 m long with 0.75 m between rows and 0.25 m within row spacing in two or three replications (Table 2). Surrounding each nest and trial were blends of male pollinator planted in adequate rows to ensure adequate pollen for the duration of silking. For locations with one leaf or one and three leaves removed, the pollen producing (PP) and non-pollen producing (NPP) single-crosses were nested side by side. For the second trial with one to five leaves removed, pedigrees were nested together and the six treatments randomly assigned to plots within the nest.

At physiological maturity, all plants in two rows per replication per entry were hand harvested, with seed weight and moisture content measured. Seed weight was adjusted to 13.5% moisture content. From 18 locations in Kenya where one top leaf had been removed (from both Trial 1 and Trial 2), a 1 kg seed sample was randomly sub-sampled from each plot for seed grading. Seed was graded by kernel width and length by passing the seed through a series of screens. Firstly, seeds were separated into width classes using round hole screens. Seed was passed successively through large (11.0 mm), medium (9.5 mm) and small (6.5 mm) screens to separate seed. Seed from each width class was separated by passing the samples though slotted screens. Seed that failed to pass through a 6.5 mm slotted screen was classified as round. Seed that passed through the 6.5 mm screen but failed to pass through a 5.5 mm slotted screen was classified as thick seed. Seed that passed through the 5.5 mm slotted screen was classified as flat. The number of seeds in each of the six classes was counted. For each class, two samples of 100 kernels each were taken, and 100-kernel weight was estimated. For the large flat and large round seed classes, the majority of plots had less than 100 kernels; thus, the data from these two classes was excluded from subsequent analysis.

### Establishing number of leaves removed during detasseling in commercial seed production fields

The number of leaves removed during detasseling was measured at three seed companies in both South Africa and Zimbabwe. In each three-way hybrid maize seed production field, detasseling teams were employed as contracted labour by seed companies. The level of training and experience varied within each team. After the detasseling teams removed the tassels from the female parent plants, 2000 tassels were collected. The detasseling teams were not informed in advance to avoid any possible bias. Tassels were transported back to the field stations and the number of top leaves attached to the tassel were counted.

### Data analysis

All analyses were conducted in R (version 4.2.2.). Yield data were analysed using linear mixed-effects modelling. The two trials were analysed separately and combined. The number of top leaves removed was treated as a continuous fixed effect to estimate the yield reduction per-leaf removed. The random effect’s structure was kept as maximal as the experimental design allowed. Each model contained a random intercept for location-year (the combination of year and location), a pedigree replicate within a location-year, and a random slope and intercept for each pedigree in a location-year. The overall effect of leaf removal on yield was then quantified by computing the (restricted) maximum likelihood (REML) estimate of the coefficient associated with number of leaves removed.

Seed grade data were analysed using logistic mixed-effects regression for kernel counts and linear mixed effects regression for kernel weight, with a fixed effect factor indicating whether seed was pollen or non-pollen producing and a random intercept for location-year, a pedigree replicate within a location-year, and for each pedigree in a location-year. Independent models were fit for each grade. The p-values were adjusted using Holm-Sidak.

### Model fitting

The fit of the joint data to the model is evaluated by checking several model assumptions including linearity of yield with respect to leaf removal, homogeneity of variance, normality of residuals, and normality of random effects (Supplementary Fig. 1). There was no egregious deviation from the assumption of linearity and homogeneity of variance. Using the Cook’s distance and a threshold of 0.7, 93 outliers were identified. After examining each data entry, none were found to be because of data entry error or due to any systematic error so none of the entries were excluded from the analysis. However, the residuals did not appear normally distributed, implying that standard inferences and p-values would be uncertain, thus a fully nonparametric bootstrap method was used to obtain p-values and confidence intervals^38^. The resampling in the bootstrap is done at the two lowest level clusters: replicates and observations using 1000 bootstrap samples.

## Supplementary Information

Supplementary Information.

## Data Availability

The data that support the findings of this study and the code for all statistical analysis and figures are available on CIMMYT Dataverse (https://hdl.handle.net/11529/10549126).
