# Supplementary material for: Ms44-SPT: unique genetic technology simplifies and improves hybrid maize seed production in sub-Saharan Africa
Source: Sci Rep. 2024 Dec 30;14:32125. doi: 10.1038/s41598-024-83931-1 (PMC11686286; doi:10.1038/s41598-024-83931-1)
Supplement: Supplementary file 1 — Supplementary Information. [file 41598_2024_83931_MOESM1_ESM.pptx]

## Slide 1
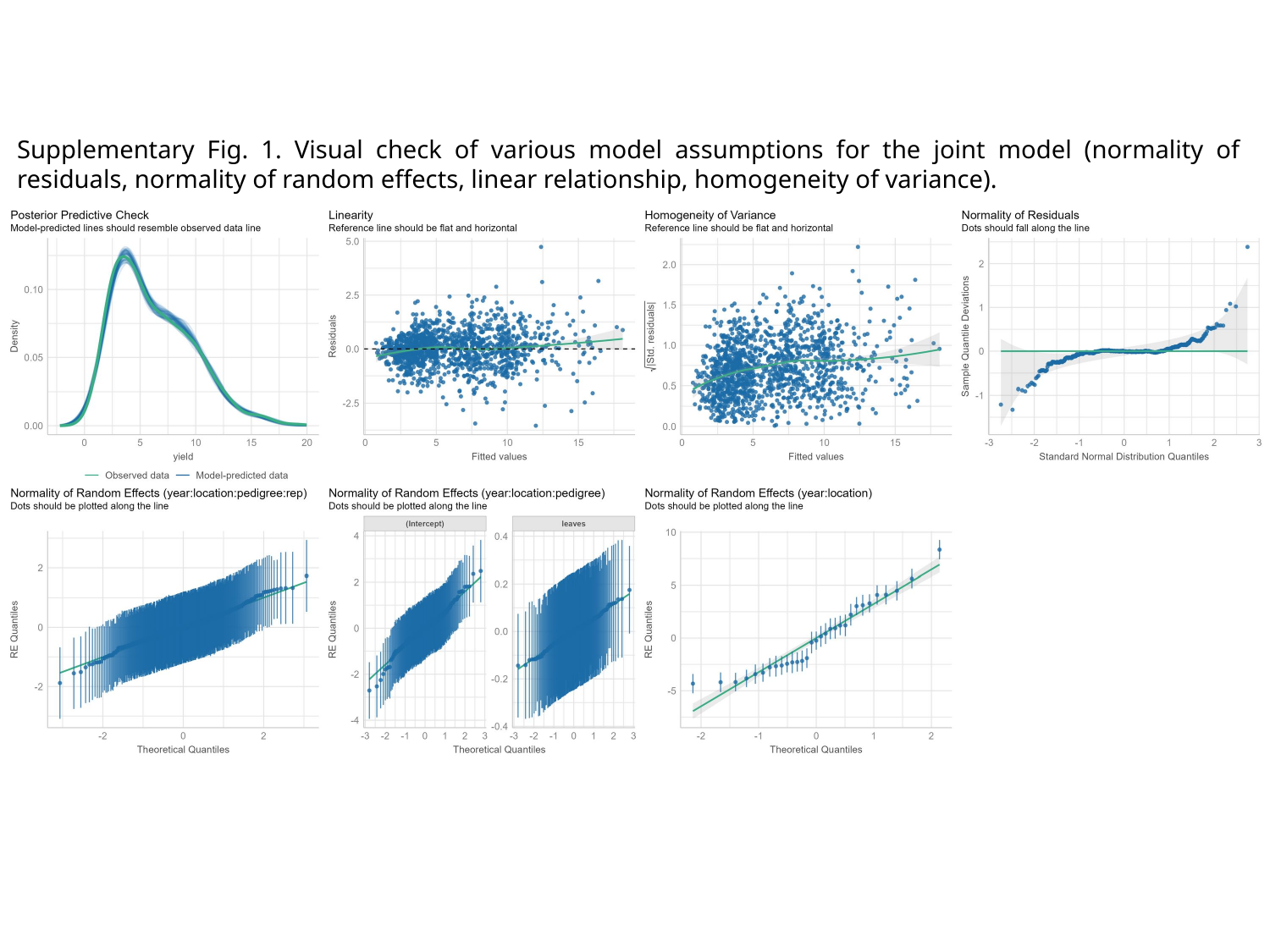

Supplementary Fig. 1. Visual check of various model assumptions for the joint model (normality of residuals, normality of random effects, linear relationship, homogeneity of variance).
